# Density Scaling Based Detection of Thermodynamic Regions of Complex Intermolecular Interactions Characterizing Supramolecular Structures

**DOI:** 10.1038/s41598-020-66244-x

**Published:** 2020-06-09

**Authors:** Sebastian Pawlus, Andrzej Grzybowski, Sławomir Kołodziej, Michał Wikarek, Marzena Dzida, Paweł Góralski, Scott Bair, Marian Paluch

**Affiliations:** 1 August Chełkowski Institute of Physics, University of Silesia in Katowice, ul. 75 Pułku Piechoty 1, 41-500 Chorzów, Poland; 2 Institute of Materials Science, University of Silesia in Katowice, ul. 75 Pułku Piechoty 1, 41-500 Chorzów, Poland; 3 Institute of Chemistry, University of Silesia in Katowice, ul. Szkolna 9, 40-006 Katowice, Poland; 4 Department of Physical Chemistry, Faculty of Chemistry, University of Lodz, ul. Pomorska 163/165, 90-236 Łódź, Poland; 5 George W. Woodruff School of Mechanical Engineering, Atlanta, GA 30332-0405 USA

**Keywords:** Condensed-matter physics, Soft materials, Self-assembly, Atomic and molecular physics, Macromolecules and clusters, Structure of solids and liquids

## Abstract

In this paper, applying the density scaling idea to an associated liquid 4-methyl-2-pentanol used as an example, we identify different pressure-volume-temperature ranges within which molecular dynamics is dominated by either complex H-bonded networks most probably leading to supramolecular structures or non-specific intermolecular interactions like van der Waals forces. In this way, we show that the density scaling law for molecular dynamics near the glass transition provides a sensitive tool to detect thermodynamic regions characterized by intermolecular interactions of different type and complexity for a given material in the wide pressure-volume-temperature domain even if its typical form with constant scaling exponent is not obeyed. Moreover, we quantify the observed decoupling between dielectric and mechanical relaxations of the material in the density scaling regime. The suggested methods of analyses and their interpretations open new prospects for formulating models based on proper effective intermolecular potentials describing physicochemical phenomena near the glass transition.

## Introduction

Investigations of intra- and intermolecular interactions characterizing different molecular structures have held a tremendous amount of attention of many researchers since the 19^th^ century. However, there are still unsatisfactorily explored issues in this extensive field. A commonly known challenge constitutes supramolecular structures and their specific interactions in various thermodynamic phases. This matter becomes especially difficult to investigate if we do not limit our study to the ambient pressure conditions, but extend it over a wide temperature-pressure range to result in relatively large variations in the density domain. In this paper, we present a novel approach to detect regions within which supramolecular structures characterized by complex specific interactions are expected in the thermodynamic space.

Since the beginning of this century, a very promising idea of the density scaling of molecular dynamics in viscous liquids has been successfully developed to make progress in the search for a complete and commonly accepted theory of the glass transition and related phenomena^1–4^. According to this idea, a dynamic quantity *Y* (e.g., viscosity, structural relaxation time or segmental relaxation time in case of polymers) measured at different temperatures *T* and pressures *p* along different isobars and isotherms can be scaled onto one master curve well described by a function *f* of the single variable Γ as follows

1 $$Y=f(\Gamma )$$

where $\Gamma ={\rho }^{\gamma }/T$ with the density ρ defined as the inverse specific volume ${V}^{-1}$ and the scaling exponent *γ* which is a material constant independent of thermodynamic conditions according to the typical viewpoint at the beginning of this idea development^5^. A significance of Eq. (1) relies on the theoretical grounds for its scaling exponent *γ* related to an exponent of the dominant repulsive part ($\approx 3\gamma$) of the effective short-range intermolecular potential derived from the well-known Lennard-Jones potential, which have been satisfactorily verified by computer simulations^6–10^. Therefore, the power density scaling (PDS) law expressed by Eq. (1) gives a tempting opportunity to study macroscopic material properties by using their underlying intermolecular potential as well as to evaluate parameters of the relevant intermolecular potential based on macroscopic quantities measured for various materials.

It should be noted that the scaling law given by Eq. (1) was called *thermodynamical scaling* by Casalini and Roland^5^. This name has been widely used by them and other authors, but almost simultaneously, another term has been proposed, i.e., *scaling with a power law in density*^11^. Later, this term has been popularized as *density scaling* or *density scaling law* by the Roskilde group, which significantly contributed to understand theoretical grounds for Eq. (1), starting from finding a strong correlation between instantaneous average virial and potential energy in simple simulations models^6–8^. According to the latter approach and earlier publications^4^, in this paper, we prefer to use the term *power density scaling law*, which invokes the power form of the density scaling function implemented in Eq. (1), and additionally enables to informatively specify whether the scaling exponent of the PDS law is invariant or not.

In many cases of materials belonging to different material groups (such as van der Waals liquids and polymer melts, but also ionic liquids, some phases of liquid crystals, and metallic glass formers), it has been experimentally validated that the PDS law is obeyed when $\gamma =const$ for a given material at least to a good approximation^12^. Thus, the PDS law bears hallmarks of universality, becoming a convenient tool to gain a better insight into complex physicochemical phenomena occurring mainly near the glass transition, but also in the normal liquid state^13^ and some liquid crystal phases^14,15^.

Excluding results of molecular dynamics simulations in mainly isotropic simple models, which do not seem to be representative for real materials usually consisted of anisotropic molecules, a few van der Waals liquids^16,17^ and several metals^17,18^ have been reported to possess scaling exponents *γ* dependent on state points or only density, but these cases are still hotly debated^19–21^. However, undoubted examples of the deviation from the PDS law with the invariant scaling exponent *γ* for a given material have been found among associated liquids^22–26^. Then, a question arises *whether the PDS law with the scaling exponent γ dependent on thermodynamic conditions for a given material may usefully inform us about some properties of the material?* To answer this question, in this paper, we perform the density scaling based analysis of supercooled monohydroxy alcohol, 4-methyl-2-pentanol (4M2P) as an example of associated liquids, and consequently provide a handy tool to identify the range of thermodynamic variables (*p*-*V*-*T*), within which complex hydrogen-bonded networks should be formed.

## Results and Discussion

To collect an experimental data set covering a wide thermodynamic range, we have performed extensive measurements of dielectric relaxations and viscosity at ambient and elevated pressure as well as the speed of sound under high pressure, density and specific isobaric heat capacity at atmospheric pressure for 4M2P. Details of the measurement techniques have been described elsewhere^27–31^ and their brief description including experimental ranges for 4M2P is presented in Methods.

It is worth noting that monohydroxy alcohols with hydroxyl group located at or close to the terminal position of the carbon chain constitute an important group of associated liquids. For these materials, chain-like H-bonded structures prevail^32^. Motions of these transient chains are source of the so-called Debye-like relaxation observed mainly in the dielectric spectra of the alcohols like e.g. 1-propanol or 2-ethyl-1-hexanol (2E1H)^33,34^. This process (exponentially broadened at elevated pressure) is slower and decoupled from the structural relaxation, although it also exhibits a non-Arrhenius temperature dependence of the relaxation times, ${\tau }_{D}$^32^. In case of 4M2P, we have also observed the same kind of dielectric relaxation (see Methods herein and Supplementary Fig. S1) along 16 isotherms and 1 isobar at ambient pressure (see Supplementary Fig. S2). It should be noted that comparison of the Debye-like relaxations on the dielectric loss spectra of 2E1H and 4M2P reveals virtually the same intensity of these processes (see Supplementary Fig. S3). It was shown^35^ that for primary alcohols the amplitudes of the Debye-like mode are very similar. This indicates the same architecture and population of H-bonded structures, in that case dominated by chain-like associates. On the other hand, for alcohols with hydroxyl group located at non-terminal position on the alkyl chain, e.g. 5-methyl-3-heptanol and 4-methyl-3-heptanol, the intensity of the Debye-like process decrease^36,37^. This is the consequence of increasing number of ring-like entities in the liquid. Since the amplitude of the Debye-like mode in 2E1H is the same as for 4M2P it can be concluded that for the latter alcohol H-bonded structures are dominated by the transient chains. With pressurization the intensity of the Debye-like mode increases, however this increase is smaller than in the material isobarically cooled at ambient pressure (see Supplementary, inset to Fig. S4). This difference is reflected by changes of dielectric strength, Δε, of the relaxation during isobaric cooling and isothermal pressurization (see Supplementary Fig. S4). Although for both thermodynamic paths Δε increases with increasing τ but for isothermal results this increase is much lower. It has to be noticed that in case of total decomposition of H-bonded structures responsible for the Debye-like process during pressurization we will expect a gradual disappearance of this relaxation. Though this is not the case here and it can be concluded that although the compressive forces modify the properties of Debye-like mode markedly, they definitely do not destroy the chain-like associated structures that are the source of this process.

To attempt to scale the Debye-like relaxation times in terms of Eq. (1), the experimental dielectric data collected in the *T*-*p* domain should be transformed to the *T*-*V* or *T*-*ρ* domain. To do that we have parametrized the dependence *V*(*T*,*p*) by fitting the pVT measurement data to the commonly known Tait equation of state^38^ to find values of its parameters shown in Methods. Then, the isothermal and isobaric dependences ${\tau }_{D}(V)$ have been established (Supplementary Fig. S5).

A convenient criterion for the density scaling expressed by Eq. (1) with $\gamma =const$ can be formulated by the following linear equation^39^,

2 $${\log }_{10}T=\gamma {\log }_{10}\rho +C\,{\rm{at}}\,Y=const$$

where the parameter *C* may depend only on the dynamic quantity *Y* and the scaling exponent *γ* is a constant slope of the log-log dependence of *T* vs *ρ* (or ${V}^{-1}$) to a good approximation. Then, the PDS law with the invariant scaling exponent *γ* is obeyed for a given material.

**Figure 1:**
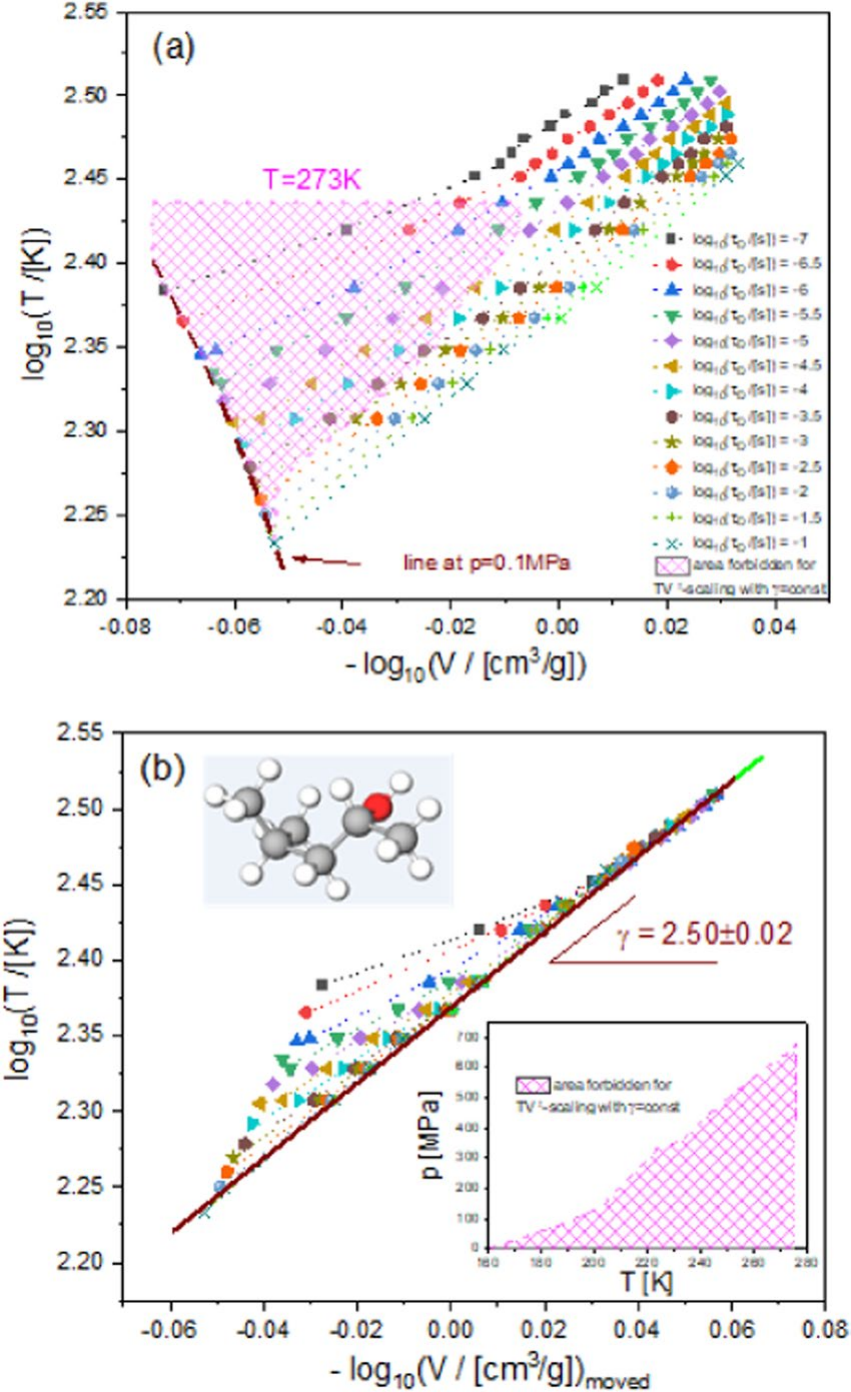
(**a**) Log-log plot of temperature vs. inverse volume at different constant Debye-like relaxation times. (**b**) Determination of density scaling exponent by horizontal shifts of the isochrones shown in panel (a) onto the isochrone at *τ*_*D*_ = 0.1 s. The shaded areas in both panels represent the *pVT* domain within which the density scaling with *γ* = *const* is not satisfied.

We have applied the PDS criterion to the experimental Debye-like relaxation times of 4M2P at ${\tau }_{D}=const$ selected every half decade in the range of ${\tau }_{D}$ between 0.1μs and 0.1 s. As can be seen in Fig. 1(a), Eq. (2) with $\gamma =const$ is not met in the entire analysis range, which includes the temperature-density regions of both the invariant and state-point dependent scaling exponent *γ*, where the latter region has been shaded. From the thermodynamic region of the linear dependences of ${\tau }_{D}$ described by Eq. (2), we have established a constant value of the scaling exponent, $\gamma =2.50\pm 0.02$, which is relatively small and similar to earlier reported values of *γ* for some H-bonded liquids (e.g. sorbitol)5 that satisfied the PDS law with $\gamma =const$ to a good approximation. To better recognize the thermodynamic region of state-point dependent scaling exponent *γ*, we have moved horizontally the isochrones shown in Fig. 1(a) to the isochrone at ${\tau }_{D}=0.1{\rm{s}}$, which is linear in the analysis range. In this way, we have illustrated the deviation from the PDS law with $\gamma =const$ in Fig. 1(b). Moreover, in the inset in Fig. 1(b), we have reconverted the *T*-*ρ* domain to the *T*-*p* domain of the analysis based on Eq. (2), showing for convenience the *T*-*p* region of state-point dependent scaling exponent *γ*.

**Figure 2:**
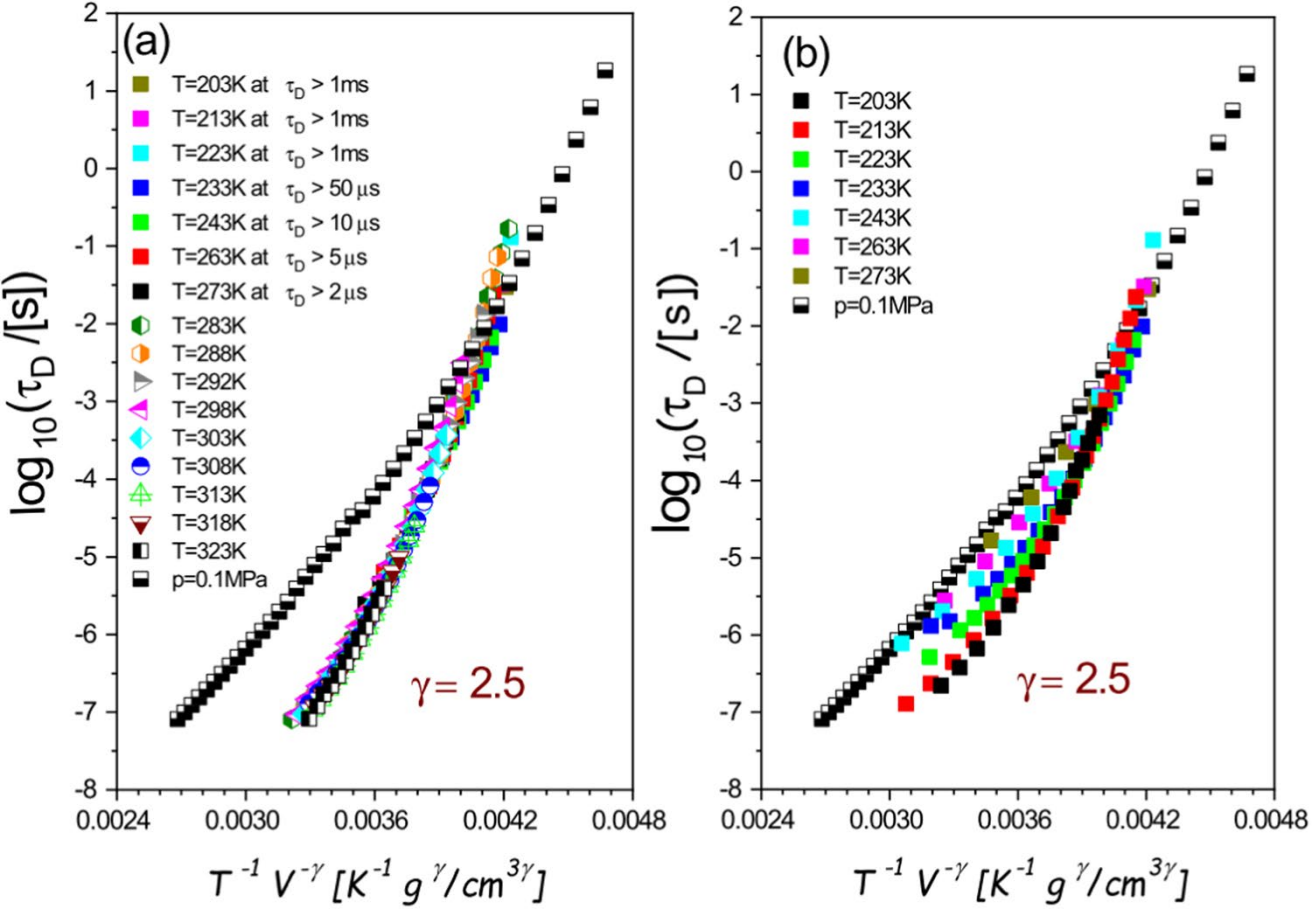
(**a**) Density scaling plot with the exponent *γ* = 2.5 after excluding parts of isotherms at low temperatures and short relaxation times according to the analysis shown in Fig. 1. The atmospheric isobar does not match the scaling pattern satisfied by isotherms at high temperatures and selected parts of isotherms at low temperatures. (**b**) Density scaling plot with the exponent *γ* = 2.5, including the atmospheric isobar and isotherms at low temperatures; such a density scaling is invalid for isotherms at low temperatures and short relaxation times.

All the analyses based on the PDS criterion (Eq. (2)), which are presented in Fig. 1, give evidence that the Debye-like relaxation times of 4M2P conform to the PDS law (Eq. (1)) with $\gamma =const$ at ${\tau }_{D} > 1\,{\rm{ms}}$ (excluding almost all points at ambient pressure), while at ${\tau }_{D}\le 1\,{\rm{ms}}$ only above some limiting values of pressure. The limiting values of *p* systematically increase (from 0.1 MPa to 650 MPa) with increasing values of temperature (from 160 K to 273 K). Correspondingly, the entire isotherms of ${\tau }_{D}$ between 283 K and 323 K can be scaled according to Eq. (1) with $\gamma =2.5$, while other isotherms of ${\tau }_{D}$ measured at temperatures between 203 K and 273 K satisfy the PDS law with this scaling exponent *γ* above some value of ${\tau }_{D}$ separately established for each of them (Fig. 2(a)). Along the demarcation line between the invariant and state-point dependent scaling exponent *γ* for the low-temperature isotherms, the values of ${\tau }_{D}$ increases with decreasing isotherm temperature from ${\tau }_{D}=2\,{\rm{\mu }}{\rm{s}}$ at T = 273 K and reaches a plateau ${\tau }_{D}\approx 1\,{\rm{ms}}$ at ${\rm{T}}\le 233{\rm{K}}$. This analysis leads to no density scaling with $\gamma =2.5$, which is presented in Fig. 2(b) for the values of ${\tau }_{D}$ below the demarcation line in case of the low-temperature isotherms. It is worth noting that Casalini and Ransom have recently shown^40^ that the scaling exponent gamma increases with increasing pressure, tending to a constant value in the high pressure limit in case of a few hydrogen bonded materials. Our results obtained for 4M2P reveal a more complex dependence of the scaling exponent on thermodynamic conditions, because we have found that the pressure dependence of the scaling exponent for 4M2P strongly changes with varying temperature. That is, as already mentioned, an increase in temperature is related to a considerable increase in the pressure limit above which the $T{V}^{\gamma }$–scaling is satisfied with $\gamma =2.5$ by the Debye-like relaxation times for 4M2P (see the inset in Fig. 1(b)).

The analyses of the thermodynamic evolution of the Debye-like relaxation times ${\tau }_{D}$ depicted in Figs. 1 and 2, which are respectively based on the PDS criterion (Eq. (2)), and then on the PDS law (Eq. (1)), show that the density scaling behavior of 4M2P is more complex than those earlier observed for associated liquids. For instance, in case of the already mentioned monohydroxy alcohol 2E1H, Fragiadakis *et al*.^41^ reported that the thermodynamic scaling is generally not valid, and later Pawlus *et al*.^25^ have shown that the PDS law with a constant $\gamma =1.8$ can be, however, successfully applied to the Debye-like relaxation times ${\tau }_{D}$, but only up to the pressure of *ca*. 0.5 GPa, above which the scaling exponent *γ* linearly increases with ${\tau }_{D}$. Thus, it is uniquely defined by the time scale of H-bonded structures rearrangements, which are reflected in the Debye-like dielectric process of 2E1H. An associated liquid *N*,*N*-diethyl-meta-toluamide (DEET), widely used as an insect repellent, reveals a more complicated dependence of the scaling exponent *γ* on the structural relaxation time τ described by a cubic function in the entire range of the density scaling based analysis. However, the monohydroxy alcohol 4M2P considered herein is characterized by two separate thermodynamic regions, within which the PDS law with $\gamma =const$ is either valid or broken, where the scaling exponent *γ* in the latter one cannot be described by any function only of the time scale ${\tau }_{D}$ as can be deduced from Fig. 1(a). Moreover, the scaling exponent *γ* cannot be also any function only of density, which was earlier suggested for two supercooled van der Waals liquids measured in the wide *T*-*p* range^16^, because it has not been possible to superimpose the nonlinear parts of isochrones in Fig. 1(b). It means that the scaling exponent *γ* for 4M2P is state-point dependent in the thermodynamic region where the PDS law is broken for this material, i.e., it varies when temperature and pressure changes.

In case of associated liquids, the deviations from the PDS law with $\gamma =const$ are most likely caused by forming complex H-bonded networks. Earlier investigations of the dynamics of H bonds under high pressure^42,43^ showed that the increasing pressure rather results in the increase in the degree of H bonds (i.e., in their strength or/and number) at least up to 1 GPa. Seemingly, the found *T*-*p* range of the state-point dependent scaling exponent *γ* for 4M2P follows a different pattern of behavior, because the limit pressure between the invariant and state-point dependent *γ* considerably changes with varying temperature (see the inset in Fig. 1(b)). However, the mentioned earlier reports on H bonds under high pressure were actually based on an analysis of the liquid fragility determined at different glass transition temperatures *T*_*g*_ at different values of pressure, where *T*_*g*_ is defined in some arbitrarily chosen isochronal conditions defined by a constant structural relaxation time. In this context, it should be noted that the isochronal compression is accompanied with an increase in temperature. This experimental fact was used to explain the increase in the degree of H bonds of propylene glycol oligomers up to *ca*. 1 GPa due to decreasing intermolecular distances under compression, and the decrease in the degree of H bonds at $p > 1\,{\rm{GPa}}$ pressure due to an increasing role of temperature fluctuations in molecular dynamics of those associated liquids. If we inspect the isochrones at ${\tau }_{D}\le 1\,{\rm{ms}}$ (see Fig. 1(a)), we can observe a very similar behavior in case of 4M2P. Along these isochrones, the thermodynamic ranges of the state-dependent scaling exponent *γ* are located at low temperatures and densities, i.e., at low temperatures and pressures, while the PDS law with $\gamma =2.5$ is obeyed above some values of temperature and density (and above some corresponding value of *p*), which depend on the time scale of isochrone. Thus, the complex density scaling of the Debye-like relaxation times of 4M2P observed at ${\tau }_{D}\le 1\,{\rm{ms}}$ can be explained by changes in the degree of H bonds due to competing effects of compression and temperature fluctuations on the H-bonded network dynamics similarly as reported for propylene glycol oligomers near the glass transition^42,43^. Analogically, the validity of the PDS law with $\gamma =2.5$ for 4M2P above ${\tau }_{D}=1\,{\rm{ms}}$ at each considered temperature, excluding pressures near the ambient conditions, suggests that the temperature fluctuations dominate the dynamics of H bonds of 4M2P at these time scales even at low temperatures. It shows that the PDS criterion (Eq. (2)) is a very sensitive tool to detect the thermodynamic regions of complex specific interactions like strong or/and numerous hydrogen bonds (if *γ* varies) and typical molecular interactions like van der Waals forces (if *γ* is invariant) for a given material.

Finally, we have performed the density scaling based analysis of viscosity *η* measured for 4M2P along an atmospheric isobar and a few isotherms up to $p=1\,{\rm{GPa}}$ in order to compare its results with the previous one for ${\tau }_{D}$. This study has been motivated by the well-known difficulties in experimental explorations of the structural dielectric relaxation in monohydroxy alcohols.

**Figure 3:**
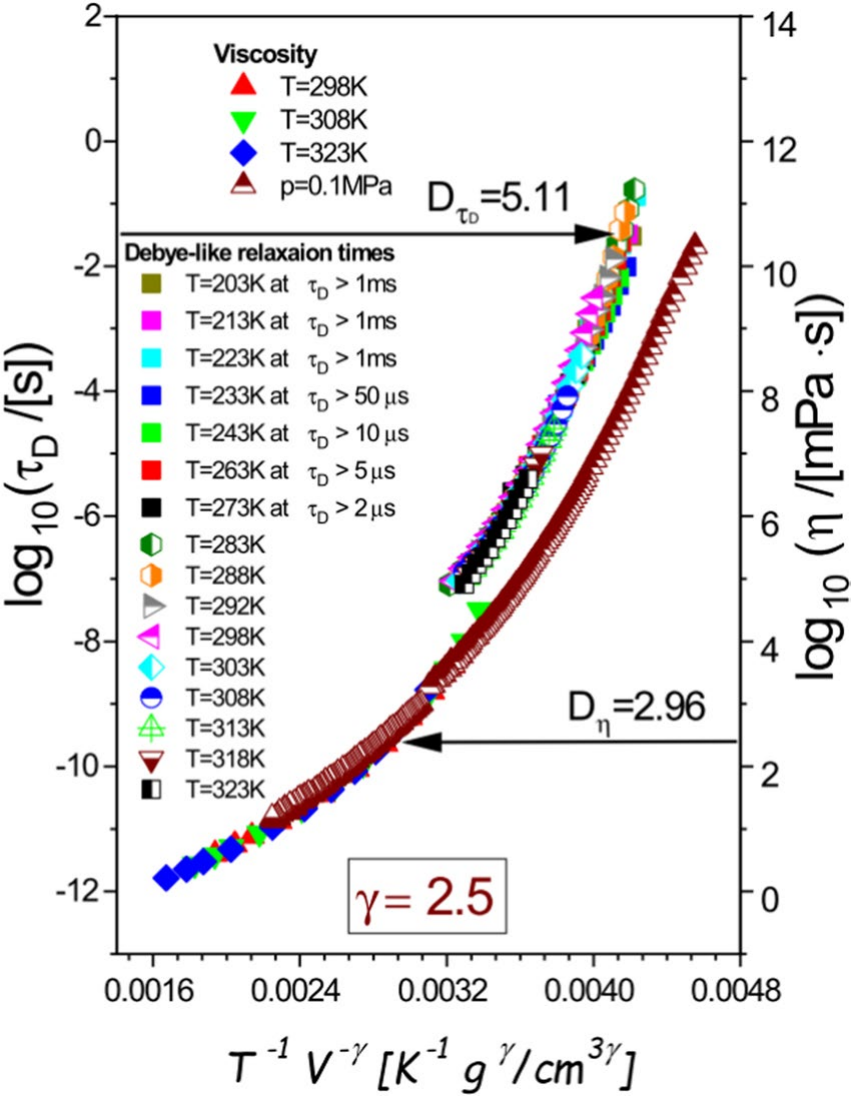
Comparison of the density scaling of viscosity and Debye-like relaxation time with *γ* = 2.5, including all viscosity data and only isothermal Debye-like relaxation times shown in Fig. 2(a). This scaling pattern is satisfied by all examined viscosity data, but the master plot for η does not match that for τ, because there is a decoupling between η and τ_D_, which can be quantified by different values D_η_ and ${D}_{{t}_{D}}$ found from fitting temperature-volume dependences of η and τ_D_ to the Avramov model.

For such materials, including 4M2P, the structural dielectric loss peak is usually covered by the dominating Debye-like dielectric loss peak at sufficiently high pressures due to a higher sensitivity of the structural process than the Debye-like relaxation to the isothermal compression^35^. All the viscosity data measured for 4M2P and shown as functions of *T*, *p*, and *V* in Supplementary Figs. S6 and S7 available has turned out to satisfy the PDS law (Eq. (1)) with the same scaling exponent $\gamma =2.5$ as that for ${\tau }_{D}$ (Fig. 3). It suggests that the same effective intermolecular potential might be relevant to the mechanical relaxation and the Debye-like dielectric relaxation in case of 4M2P at least in the thermodynamic region of the invariant scaling exponent *γ* for ${\tau }_{D}$. Nevertheless, the Debye-like relaxation times and viscosity data superimpose on two different scaling curves, which indicate a decoupling between ${\tau }_{D}$ and *η* for this material. To quantify this decoupling, we apply the Avramov model version^44–46^ that conforms to the PDS law with $\gamma =const$ (Eq. (1)), which can be formulated for a dynamic quantity, $Y={Y}_{0}\exp [{(A{\rho }^{\gamma }/T)}^{D}]$, where *Y*_*0*_, *A*, *D*, and *γ* are its parameters. By fitting separately the Debye-like relaxation times determined in the region of the invariant scaling exponent *γ* and all the measured viscosity data to the Avramov model, we have confirmed the value $\gamma =2.50\pm 0.02$ for 4M2P in case of ${\tau }_{D}$ and *η*, and respectively for these quantities, we have found different values of its other fitting parameter, ${D}_{{\tau }_{D}}=5.11\pm 0.02$ and ${D}_{\eta }=2.96\pm 0.02$. By dividing the Avramov equations applied to ${\tau }_{D}$ and *η* with the same value of *γ*, one can show that a decoupling between these quantities can be quantified by the ratio ${D}_{{\tau }_{D}}/{D}_{\eta }$ different from unity as it is for 4M2P $({D}_{{\tau }_{D}}/{D}_{\eta }\approx 1.73)$. The decoupling between *η* and ${\tau }_{D}$ accompanied with the same scaling exponent *γ* for these quantities is expected to result in the same exponent of the repulsive part of the effective intermolecular potentials relevant to the mechanical and Debye-like dielectric relaxations of 4M2P, but other parameters of the effective potentials may differ. Whereas the thermodynamic region of the state-point dependent scaling exponent *γ* observed only for ${\tau }_{D}$ shows that the dielectric spectroscopy enables to probe selectively the complex dynamics of H-bonded networks, which seems to be covered by other mechanical relaxation modes or incompletely contribute to the structural mechanical relaxation.

## Summary and Conclusions

In summary, it should be emphasized that the density scaling criterion (Eq. (2)) has turned out to be a powerful tool to investigate relevant effective intermolecular potentials based on measurements of macroscopic dynamic quantities and *vice versa* independently of the validity of the PDS law (Eq. (1)) with $\gamma =const$. The density scaling criterion applied to detect thermodynamic regions within which molecular dynamics is dominated by either typical or complex specific intermolecular interactions, depending on the invariant or varying values of *γ* for a given material, should considerably facilitate to work out intermolecular potentials to properly and effectively model various physical phenomena near the glass transition. Additionally, the suggested comparative analysis of the density scaling functions for different dynamic quantities for a given material enables to identify possible differences in the thermodynamic evolution of these quantities in the wide *pVT* range and may give hints not only on the exponent of the repulsive part of the effective intermolecular potential but also on its other parameters. Our findings open new prospects for gaining a better insight into possible changes in the character and complexity of intermolecular interactions in various materials, not only associated liquids, depending on thermodynamic conditions.

## Methods

Broadband dielectric spectroscopy (BDS) extensive studies of 4M2P have been conducted at ambient and elevated pressure. In ambient conditions, the BDS system equipped with the Alpha impedance analyzer and Quatro Cryosystem by Novocontrol GmBH have been employed in the frequency range from 10μHz up to 3 MHz at temperatures from 150 K up to 323 K. High pressure isothermal dielectric measurements have been performed in the pressure range from 0.1 MPa up to 1.8 GPa (varying depending on isotherms) and in the temperature range from 203 K up o 323 K, using two pressure systems described elsewhere^27^. Similarly to other monohydroxy alcohols with hydroxyl group located at the terminal position of the carbon chain (see e.g. refs. ^32,33^), we have observed the so-called Debye-like relaxation at elevated pressure (or the Debye process at ambient pressure) in case of 4M2P (see Supplementary Fig. S1), which has been measured along 16 isotherms and 1 atmospheric isobar. It should be noted that whereas at ambient pressure two relaxations can be discerned and the dominating one has an exponential shape (see the panel (a) in Supplementary Fig. S1), with compression both processes approach and only a single stretched Debye-like relaxation is observed. The relaxation times for this dominating process were estimated from the maximum of the relaxation curve (see black arrows in Supplementary Fig. S1). The obtained dependences of the Debye-like relaxation time ${\tau }_{D}$ on temperature at ambient pressure and pressure at constant temperatures are shown respectively in the panels (a) and (b) of Supplementary Fig. S2.

The 4-methyl-2-pentanol **(**min. 0.99 mass fraction, water content less than 2·10^−4^ mass fraction) was from Alfa Aesar. Based on the experimental speed of sound under high pressures, density and specific isobaric heat capacity at atmospheric pressure, the *pρT* data and related quantities were estimated for pressures up to 100 MPa at temperatures from 293.15 to 318.15 K by the acoustic method. The details concerning the experimental and estimation methods can be found in the previous works^30,31^. The measurement temperature-volume dependence of the specific volume *V*(*T*,*p*), where $V={\rho }^{-1}$, has been parametrized by fitting the *pVT* experimental data to the commonly known Tait equation of state^38^, $V(T,p)=({A}_{0}+{A}_{2}T+{A}_{2}{T}^{2})\{1-C\,\mathrm{ln}\,[1+p/({b}_{0}\exp (\,-\,{b}_{1}T))]\}$. The following values of the Tait equation parameters have been obtained for 4M2P, *A*_*0*_ = (1.2157 ± 0.0002) cm^3^/g, *A*_*1*_ = (1.14 ± 0.01)∙10^−3^cm^3^/(g ^◦^C), *A*_*2*_ = (2.87 ± 0.11)∙10^−6^cm^3^/(g ^°^C^2^), *C* = 0.0837 ± 0.0002, *b*_*0*_ = (103.8 ± 0.4) MPa, and *b*_*1*_ = (7.56 ± 0.05)∙10^−3◦^C^−1^. Then, the isobaric and isothermal dependences ${\tau }_{D}(V)$ have been established (see Supplementary Fig. S5).

The viscosity has been measured in falling cylinder viscometers^28,29^ to pressure of 1 GPa at three temperatures, 298 K, 308 K, and 323 K. A linear variable differential transformer monitors the sinker position rather than detecting the end of fall by electrical contact. Estimated uncertainties are 3% for viscosity, 0.5 °C for temperature and the greater of 1 MPa and 0.4% for pressure. The obtained dependences of viscosity η on temperature at ambient pressure and pressure at constant temperatures are shown in the panels (a) and (b) of Supplementary Fig. S6, respectively. Using the aforementioned parametrized dependence *V*(*T*,*p*), the isobaric and isothermal dependences of viscosity have been expressed by a function of volume (see Supplementary Fig. S7).

## Supplementary information

Supplementary information.
