# Supplementary material for: Density Scaling Based Detection of Thermodynamic Regions of Complex Intermolecular Interactions Characterizing Supramolecular Structures
Source: Sci Rep. 2020 Jun 9;10:9316. doi: 10.1038/s41598-020-66244-x (PMC7283260; doi:10.1038/s41598-020-66244-x)
Supplement: Supplementary file 1 — Supplementary information. [file 41598_2020_66244_MOESM1_ESM.pdf]

Density Scaling Based Detection of Thermodynamic Regions of Complex Intermolecular Interactions Characterizing Supramolecular Structures

Sebastian Pawlus[1,*], Andrzej Grzybowski[1], Sławomir Kołodziej[2], Michał Wikarek[1], Marzena Dzida[3], Paweł Góralski[4], Scott Bair,[5] and Marian Paluch[1]

[1] Institute of Physics, University of Silesia, ul. 75 Pułku Piechoty 1, 41-500 Chorzów, Poland.

[2] Institute of Materials Science, University of Silesia, ul. 75 Pułku Piechoty 1, 41-500 Chorzów, Poland.

[3] Institute of Chemistry, University of Silesia, ul. Szkolna 9, 40-006 Katowice, Poland

[4] Department of Physical Chemistry, Faculty of Chemistry, University of Lodz, ul. Pomorska 163/165, 90-236 Łódź, Poland

[5] George W. Woodruff School of Mechanical Engineering, Atlanta, GA 30332-0405, USA

* Corresponding author's e-mail: sebastian.pawlus@us.edu.pl

Supplementary Figures

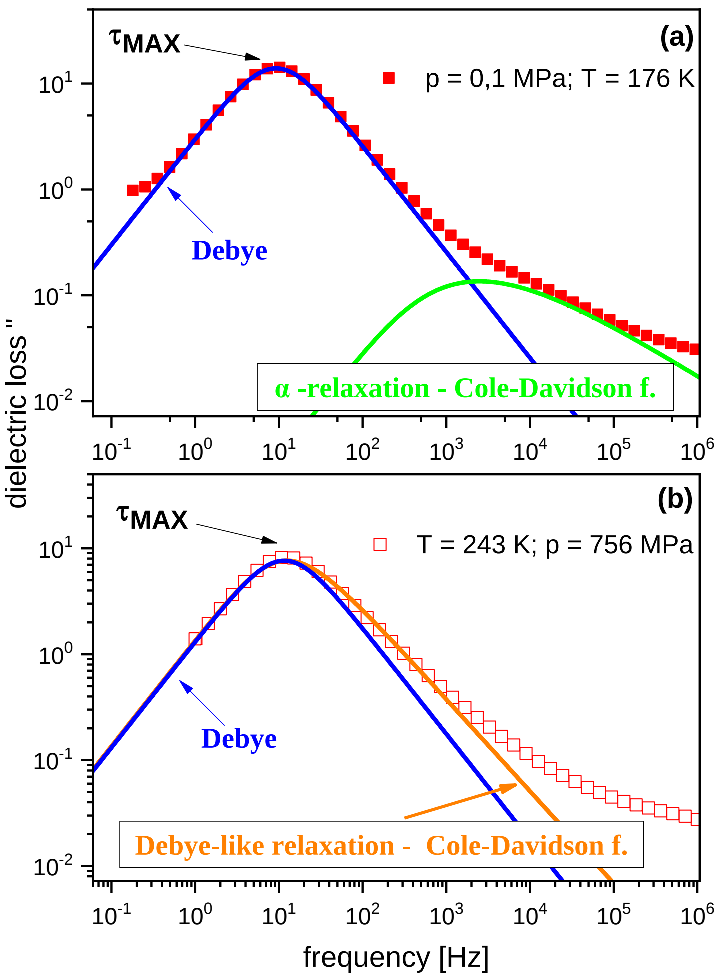

Supplementary Figure S1

Dielectric loss spectra for 4-methyl-2-pentanol collected (a) at ambient pressure and (b) in high pressure conditions. Solid curves represent different relaxation process observed in this material.

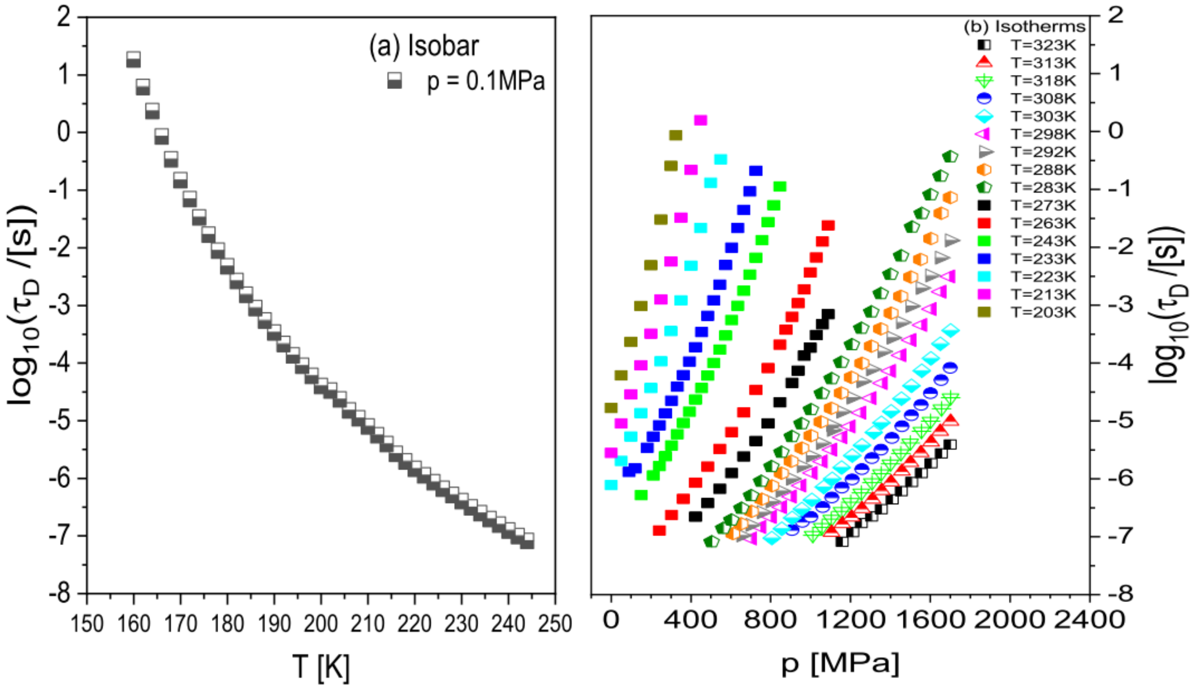

Supplementary Figure S2

(a) Temperature dependence of Debye-like relaxation times for 4-methyl-2-pentanol at ambient pressure. (b) Pressure dependences of Debye-like relaxation times for 4-methyl-2-pentanol at different constant temperatures.

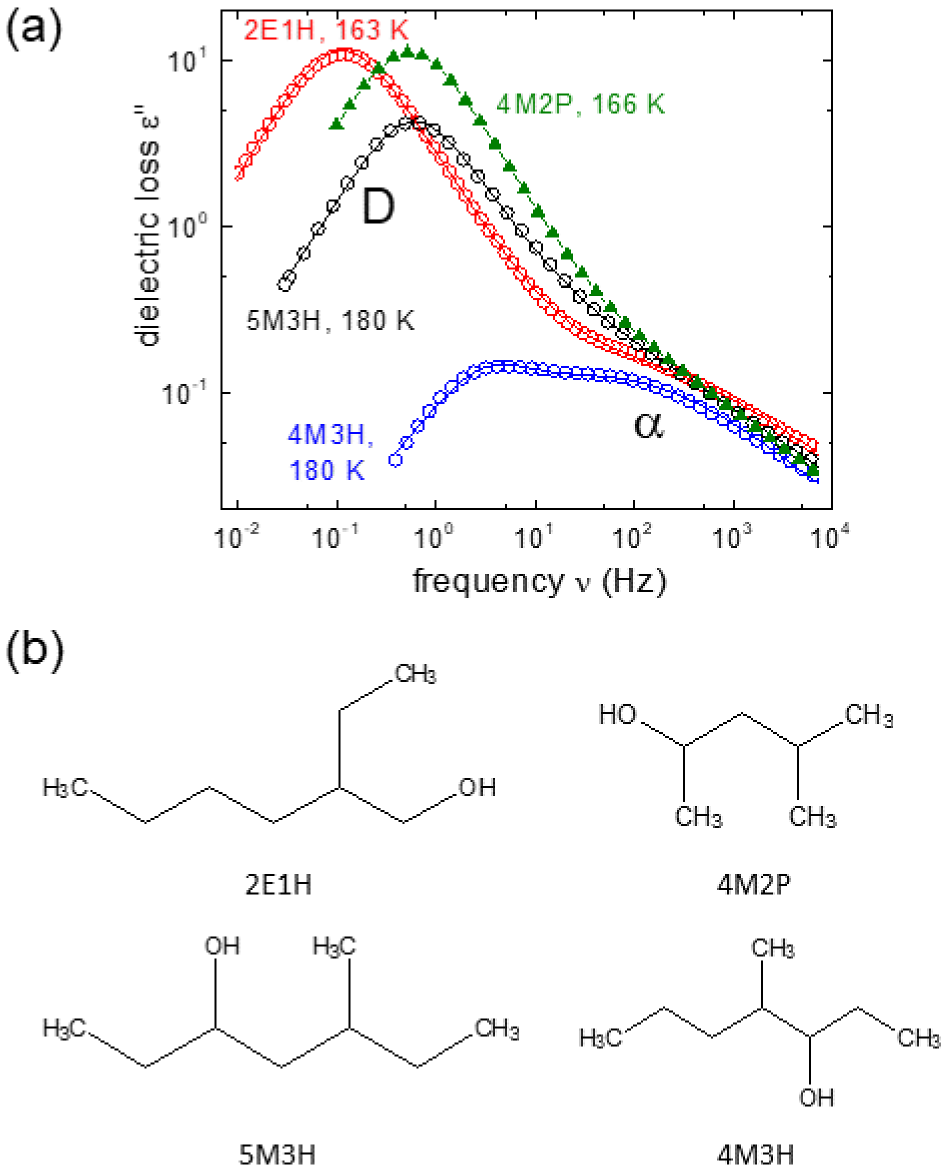

Supplementary Figure S3

(a) Comparison of dielectric loss spectra of 2-ethyl-1-hexanol (2E1H), 4-methyl-2-pentanol (4M2P), 5-methyl-3-heptanol (5M3H) and 4-methyl-3-heptanol (4M3H) registered at ambient pressure with the structural α-relaxation registered at the same frequency. (b) Structure of the alcohol molecules with respect to the nomenclature from (a).

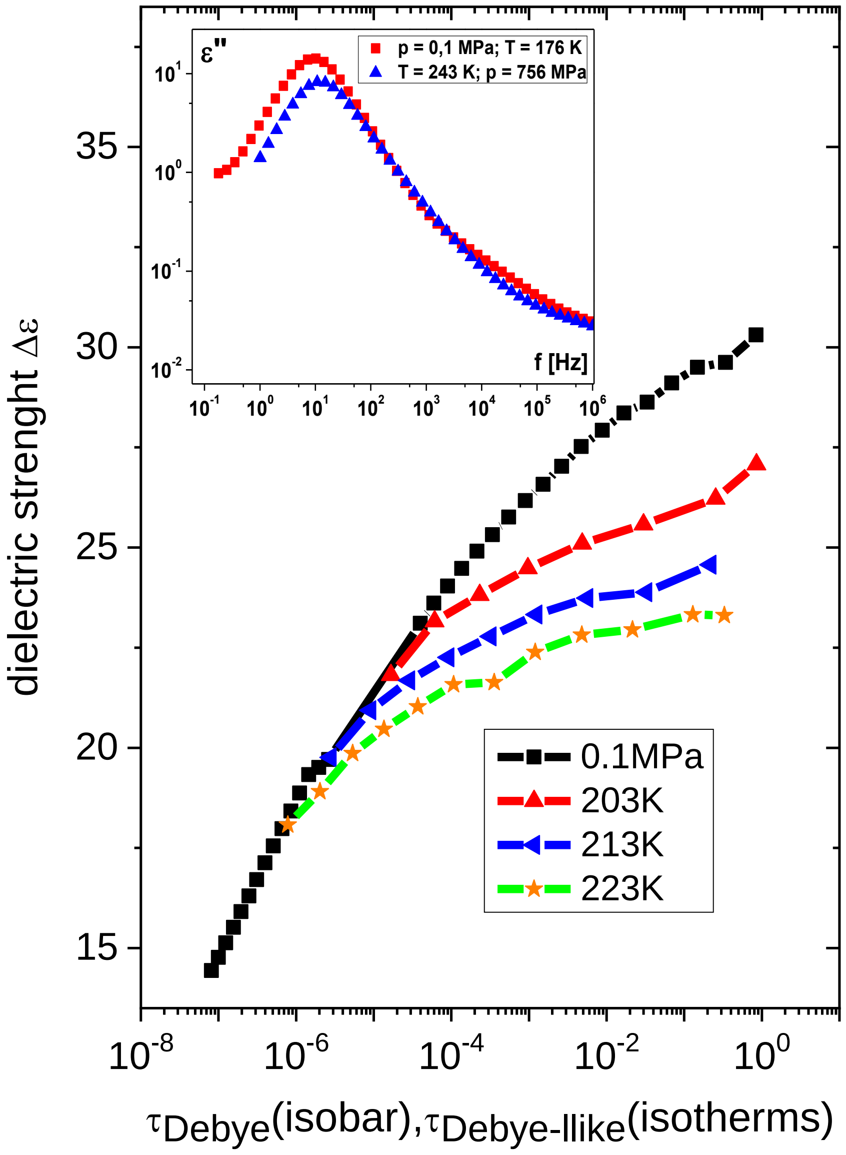

Supplementary Figure S4

Relation of dielectric strength Δε to the relaxation time of the Debye (for isobaric measurements) or Debye-like (for isothermal measurements) process for 4-methyl-2-pentanol. In inset: comparison of dielectric loss spectra of measured at ambient and elevated pressure.

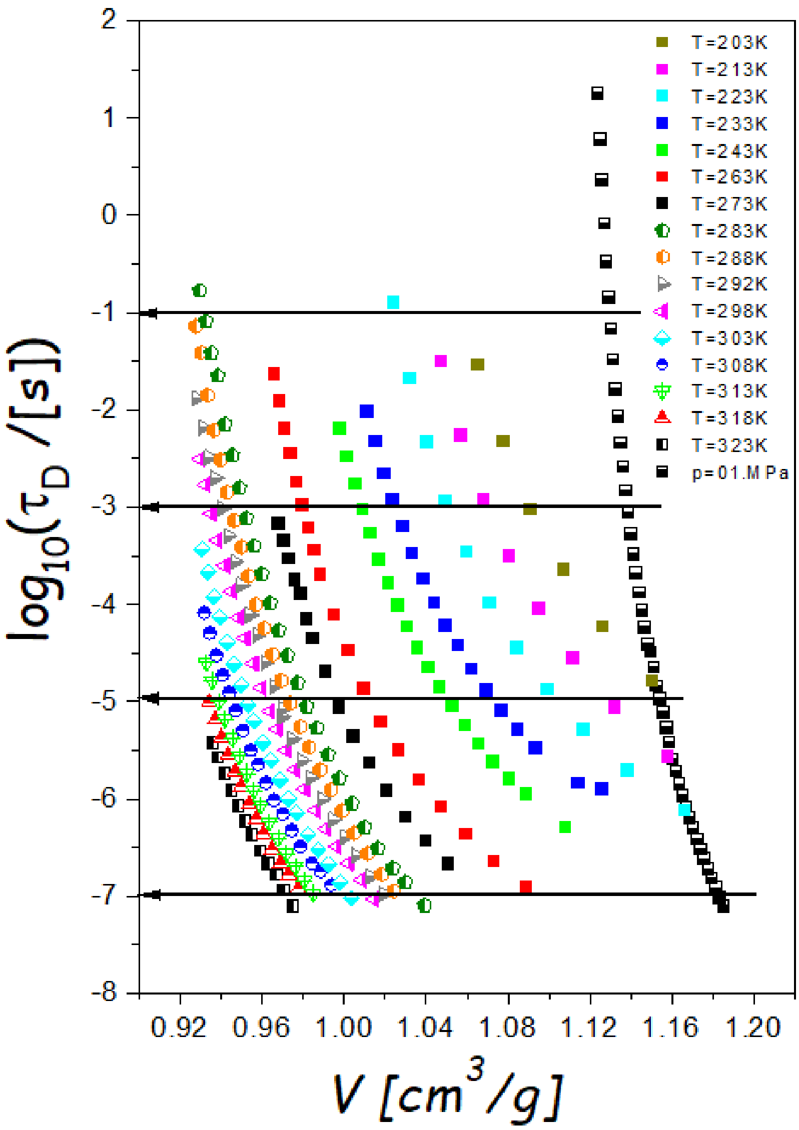

Supplementary Figure S5

Volume dependences of Debye-like relaxation times for 4-methyl-2-pentanol. Horizontal arrows indicate selected values of relaxation times.

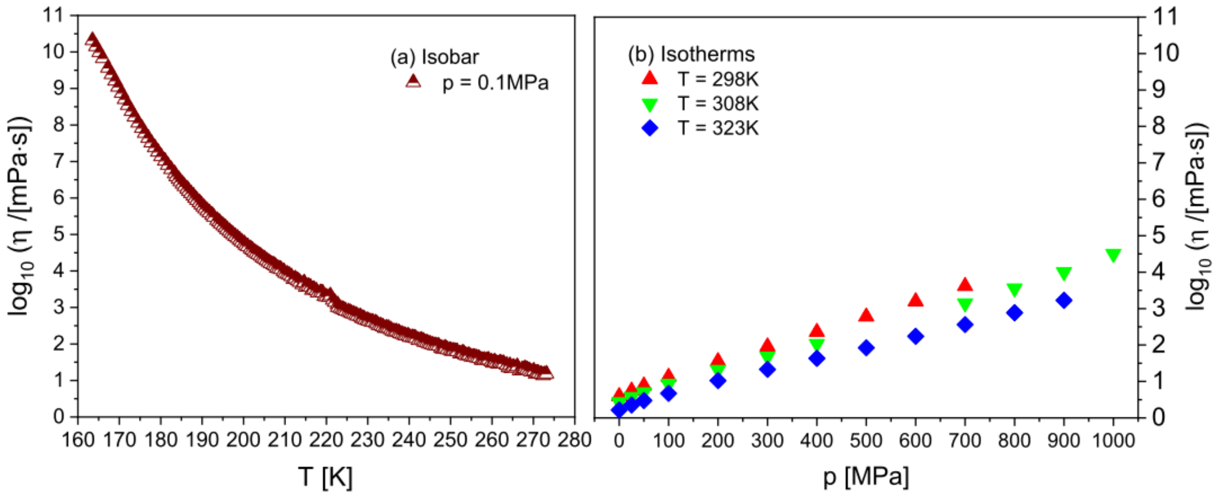

Supplementary Figure S6

(a) Temperature dependence of viscosity for 4-methyl-2-pentanol at ambient pressure. (b) Pressure dependences of viscosity for 4-methyl-2-pentanol at a few constant temperatures.

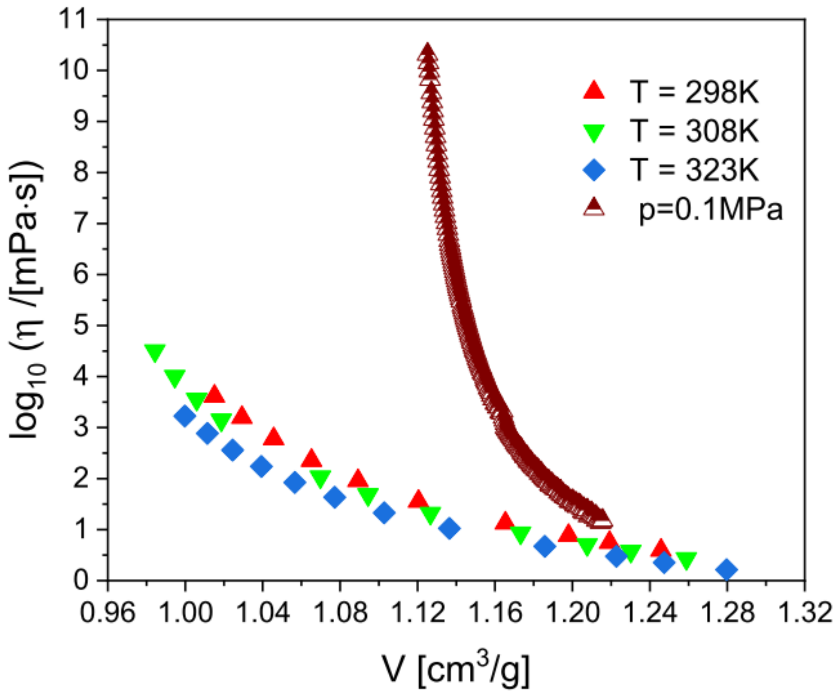

Supplementary Figure S7

Volume dependences of viscosity for 4-methyl-2-pentanol.
